# Optimal timing and mode of planned birth for term, large infants: a retrospective, population-based cohort study

**DOI:** 10.1016/j.eclinm.2025.103366

**Published:** 2025-07-17

**Authors:** Georgia Anne Santomartino, Kylie Crawford, Jesrine Hong, Sailesh Kumar

**Affiliations:** a Mater Research Institute, University of Queensland, Level 3, Aubigny Place, Raymond Terrace, South Brisbane, Queensland, 4101, Australia; b The University of Queensland School of Medicine, Herston, Queensland, 4006, Australia; c Department of Obstetrics and Gynecology, Faculty of Medicine, Universiti Malaya, Kuala Lumpur, 50603, Malaysia; d NHMRC Centre for Research Excellence in Stillbirth, Mater Research Institute, University of Queensland, Brisbane, Queensland, Australia

**Keywords:** Pregnancy, Large for gestational age, Macrosomia, Induction of labor, Caesarean section, Perinatal mortality, Neonatal morbidity

## Abstract

**Background:**

Large infants (birthweight > 75th centile) are at increased risk of mortality, severe neonatal neurological and non-neurological morbidity. We aimed to ascertain the optimal method and gestation of planned birth (scheduled caesarean section or induction of labor) that were associated with lower odds of adverse outcomes.

**Methods:**

This was a retrospective cohort study of term singleton births with birthweight >75th centile between January 2000 and December 2021 in Queensland, Australia. Primary outcomes were severe adverse maternal outcome, perinatal mortality (intrapartum stillbirth or neonatal death), severe neonatal neurological morbidity, and other severe neonatal morbidity. Multivariable logistic regression models were built to determine odds ratios (OR) for the effect of timing of both methods of planned birth on adverse outcomes. Induction of labor at 38^+0^–38^+6^ weeks was the referent category because many international guidelines recommend this as the optimum timing of birth.

**Findings:**

There were 151,464 planned births for large infants. 86,515 (57.1%) were induction of labor while 64,949 (42.9%) were scheduled caesarean section. Compared to induction of labor at 38^+0^–38^+6^ weeks, induction at ≥41^+0^ weeks (aOR 1.28, 95% CI 1.21, 1.35) and scheduled caesarean section at 37^+0^–37^+6^ weeks (aOR 1.18, 95% CI 1.08, 1.28) were associated with greater odds of severe adverse maternal outcome, whilst scheduled caesarean section at 39^+0^–39^+6^ weeks (aOR 0.75, 95% CI 0.70, 0.80) was associated with lower odds of this outcome. The odds of severe neonatal neurological morbidity were lower following induction at 40^+0^–40^+6^ weeks (aOR 0.72, 95% CI 0.59, 0.89) or scheduled caesarean section at 37^+0^–37^+6^ weeks (aOR 0.59, 95% CI 0.43, 0.81), 39^+0^–39^+6^ weeks (aOR 0.26, 95% CI 0.2, 0.33), and ≥41^+0^ weeks (aOR 0.31, 95% CI 0.13, 0.75) respectively. For other severe neonatal morbidity, the odds were highest after induction of labor at 37^+0^–37^+6^ weeks (aOR 1.35, 95% CI 1.24, 1.46), and lowest following scheduled caesarean section at 40^+0^–40^+6^ weeks (aOR 0.31, 95% CI 0.26, 0.36). There were no significant differences in perinatal mortality based on method of planned birth or gestational age.

**Interpretation:**

In our cohort, scheduled caesarean section between 39^+0^–39^+6^ weeks for large infants at birth was associated with lower odds of severe adverse maternal outcomes, severe neonatal neurological morbidity, and other severe neonatal morbidity compared to induction of labor at 38^+0^–38^+6^ weeks. For women that underwent induction of labor, the odds of emergency caesarean section were lowest at 39^+0^–39^+6^ weeks. Infants with birthweight >97th centile for gestational age had the highest risk of adverse outcomes regardless of gestation or method of planned birth.

**Funding:**

10.13039/501100000925National Health and Medical Research Council and 10.13039/100015471Mater Foundation.

**Research in context**

**Evidence before this study**

We searched PubMed for articles published from inception to 7 February 2025 with search terms “large for gestational age”, “suspected macrosomia”, “perinatal mortality”, “neonatal morbidity”, “maternal mortality”, “maternal morbidity”, “induction of labor”, “caesarean section”, “shoulder dystocia”, “brachial plexus injury”, and “perineal trauma” in various combinations with no language restrictions. Although there is evidence that rates of severe neonatal morbidity and adverse maternal outcome are lower with induction of labor at 39^+0^–39^+6^ weeks for large infants with birthweight >90th centile, the effect of method of planned birth (induction of labor vs. scheduled caesarean section) and timing of birth on these outcomes are yet to be determined.

**Added value of this study**

Our study compared maternal and neonatal adverse outcomes by method of planned birth and gestational age for large infants with birthweight >75th centile for gestational age at different birthweight centile categories. Scheduled caesarean section from 39^+0^ weeks was associated with the lowest odds of severe adverse maternal outcomes, severe neonatal neurological morbidity and other severe neonatal morbidity when compared to induction of labor at 38^+0^–38^+6^ weeks, with the lowest probabilities seen in the scheduled caesarean cohort regardless of birthweight centile category.

**Implications of all the available evidence**

Our results suggest that planned birth at 39^+0^–39^+6^ weeks is associated with better maternal and neonatal outcomes compared to induction of labor at 38^+0^–38^+6^ weeks. However, the use of any retrospective dataset is potentially limited by effects of temporal variations, unmeasured confounding or selection bias. If future studies (ideally incorporating ultrasound derived estimated fetal weight) confirm these findings then these data may help inform clinicians about optimal timing and method of planned birth for large infants.

## Introduction

Large infants are normally described as being either large for gestational age (birthweight or estimated fetal weight ≥90th centile for gestational age and sex),^1^, ^2^, ^3^ or macrosomic, variably defined as birthweight >4000 g or 4500 g.^1^^,^^2^^,^^4^ Whilst these infants make up only 6–10% of all births,^5^ their numbers are increasing globally.^6^^,^^7^ In Australia, approximately 1.2% of all infants have a birth weight of ≥4500 g.^8^ There is a strong correlation between fetal size and in-utero growth with maternal diabetes mellitus and obesity.^9^, ^10^, ^11^ From a clinical perspective, although the risks of adverse outcomes are greatest when birthweight exceeds the 95th centile for gestation, there is a clear rise in the incidence of complications when birthweights exceed the 75th centile for gestation.^12^

Infants with birthweights >75th centile are at increased risk of mortality, severe neonatal neurological and non-neurological morbidity, largely because of maternal diabetes but also because of intrapartum complications during attempted vaginal birth.^12^, ^13^, ^14^ The most recent Cochrane review^15^ showed that although induction of labor for suspected fetal macrosomia resulted in lower mean birthweight, fewer cases of shoulder dystocia, and birth fractures, there was no clear effect on the risk of caesarean section or instrumental vaginal birth, brachial plexus injury, low 5-min Apgar score or low cord arterial pH. Conversely, the 2021 National Institute of Health and Clinical Excellence Guidelines^16^ states that practitioners need to balance the reduced risk of shoulder dystocia associated with induction of labor, against the increased risks of third and fourth degree tears with such an intervention. In practice, many women opt for scheduled caesarean section in the setting of a large infant, simply to obviate the possible risks with vaginal birth.

Against this background, the aims of this study were firstly to determine the optimal timing of induction of labor associated with reduced risks of adverse maternal and perinatal outcomes and secondly to ascertain if scheduled caesarean section was associated with a lower risk of adverse outcomes compared to induction of labor for infants with birthweight >75th centile for gestation.

## Methods

### Study population

This was a retrospective cohort study of term, singleton births between 2000 and 2021 in Queensland, Australia. The dataset used for analysis was provided by the Statistical Output and Reporting Unit, Statistical Services Branch of Queensland's Department of Health and contains deidentified pregnancy data of all births in Queensland. Infants with major structural abnormalities or known genetic or chromosomal abnormalities, births <37^+0^ weeks or with no recorded gestational age, birthweight ≤75th centile,^17^ multiple pregnancy and antepartum stillbirths were excluded.

### Ethics

We used the Queensland Perinatal Data Collection^18^ with ethical and waiver of consent (as all data was anonymized) approvals granted by the Metro North Hospital and Health Service Human Research Ethics Committee (Reference number: LNR/219/QRBC/53154).

### Exposure

The effect of timing of birth and method of planned birth (induction of labor or scheduled caesarean section) on study outcomes was assessed by considering gestational age at delivery and method of birth as a 10-level categorical variable, considering both induction of labor and scheduled caesarean section at gestational ages 37^+0^–37^+6^ weeks, 38^+0^–38^+6^, 39^+0^–39^+6^ weeks, 40^+0^–40^+6^ weeks, and ≥41^+0^ weeks. For women attempting a vaginal birth the impact of gestational week on outcomes was assessed as a 5-level categorical variable between 37^+0^–37^+6^ weeks, 38^+0^–38^+6^, 39^+0^–39^+6^ weeks, 40^+0^–40^+6^ weeks, and ≥41^+0^ weeks. Induction of labor at 38^+0^–38^+6^ weeks was the referent category for both analyses because many international guidelines recommend this as the optimum timing of birth.^2^^,^^19^^,^^20^

### Outcomes

There were four primary outcomes–(1) Severe adverse maternal outcome (defined as a composite of maternal death, transfer to intensive care unit, post-partum hemorrhage >1 L, post-partum sepsis, maternal length of stay >5 days and 3rd or 4th degree perineal tears), (2) Perinatal mortality (defined as intrapartum stillbirth or neonatal death), (3) Severe neonatal neurological morbidity (defined as neonatal encephalopathy, seizures, and intracranial hemorrhage), and (4) Other severe neonatal morbidity (defined as sepsis or necrotizing enterocolitis, severe birth trauma, severe hypoglycemia, and admission to the neonatal intensive care unit or special care nursery).^21^ Severe birth trauma was defined as birth injury to head, eyes, and face, skull, long bone, clavicle, and other bone fractures, nerve injuries including brachial plexus injury and spinal cord, and birth trauma to liver, spleen and external genitalia (ICD-10 codes listed in Supplementary Table S1). Outcomes were considered mutually exclusive to enable comparability of effect sizes. Thus, an infant that died is not considered to be at risk of severe neurological morbidity and one with neurological morbidity is not considered at risk of other morbidity. Only pregnancies that had a live infant at the start of labor or before a scheduled caesarean section were included in the analysis.

Secondary outcomes (for women attempting vaginal birth) were (1) Operative vaginal birth, (2) Emergency caesarean section, (3) Emergency operative birth (caesarean section or operative vaginal birth) for failure to progress, (4) Prolonged second stage of labor (>3 h), (5) Shoulder dystocia, and (6) Severe perineal trauma (3rd or 4th degree perineal tears).

### Confounders and effect measure modifiers (interactions)

Confounders were selected based on clinical relevance^22^^,^^23^ and included maternal age, country of birth, Indigenous status, smoking, illicit drug use, alcohol use, low socio-economic status, body mass index, parity, previous stillbirth, previous caesarean section, antepartum hemorrhage, assisted conception, second stage labor >3 h, diabetes mellitus, hypertension, presentation, birthweight centile, and infant sex. Maternal health characteristics and labor outcomes were determined using ICD-10 codes (Supplementary Table S1). Socio-economic status was ascertained using the socioeconomic index for areas (SEIFA) score. The SEIFA score is generated by the Australian Bureau of Statistics and ranks Australian geographical regions according to socioeconomic advantage based on information from the five-yearly census.^24^ It defines deprivation as a SEIFA score in the lowest quintile. Birthweight centile was considered a potential effect measure modifier on timing and method of planned birth.

### Missing data

Missing values were imputed using multivariate imputation using chained equations.^25^ All missing values were imputed with intrapartum stillbirth, neonatal death, severe neurological morbidity, other severe morbidity, and method of planned birth as covariates.

### Statistics

#### Multivariable models

Multivariable logistic regression models were built to determine Odds Ratios (OR) and 95% Confidence Intervals (95% CI) for the effect of timing of planned birth on the primary and secondary study outcomes. Models were built using forward selection of all clinically relevant confounders, followed by stepwise backward elimination. Interaction terms were not included in the models due to lack of significance. Robust standard errors were used to account for clustering at the maternal level because new pregnancies in the same woman are assigned new identification numbers in the Queensland Perinatal Dataset. Marginal probabilities of adverse outcomes were then calculated and presented according to gestational age at birth and birthweight centile category. Data were analyzed using Stata 18® (Statacorp, College Station, TX, USA). This study adheres to the Strengthening the Reporting of Observational Studies in Epidemiology (STROBE) statement.^26^

### Role of funding source

The funders had no role in study design, data collection, analyses of the data, interpretation, writing of the manuscript or the decision to submit.

## Results

**Fig. 1:**
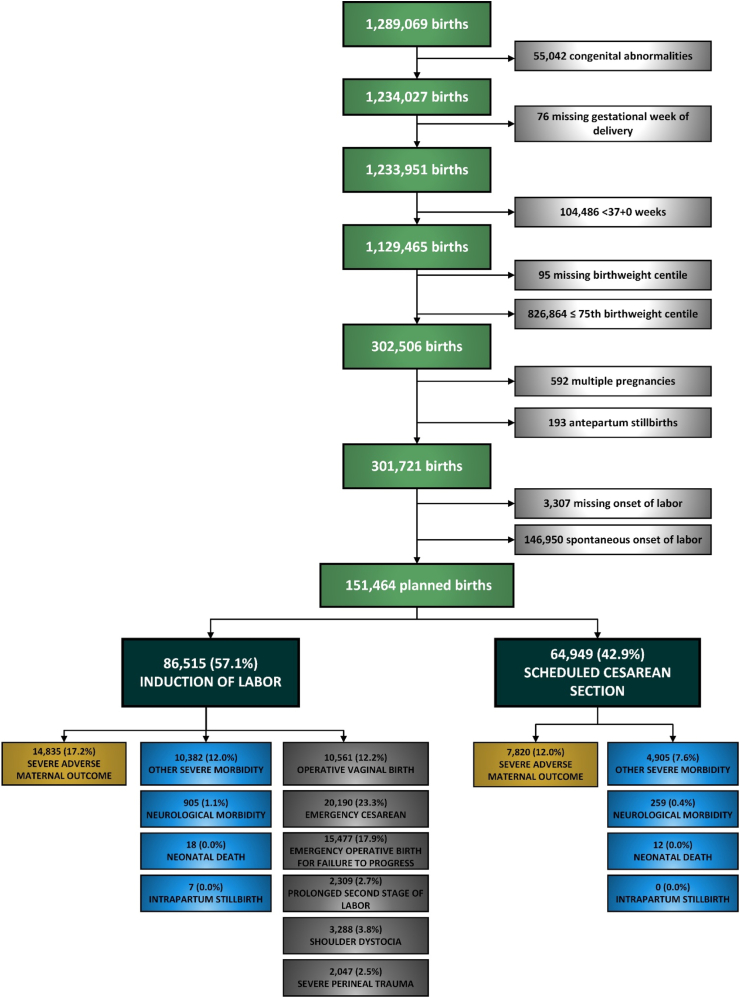
Study flow chart.

Between 2000 and 2021, there were 1,289,069 births in Queensland. After excluding preterm births, those with major structural anomalies, chromosome or genetic abnormalities, antepartum stillbirths, multiple pregnancy, birthweight ≤75th centile, women with spontaneous labor, and births with missing data for gestational age at birth, missing birthweight and onset of labor, the final study cohort comprised 151,464 women with singleton infants with birthweight >75th centile that birthed following scheduled caesarean section or induction of labor (Fig. 1). Of these, 86,515 (57.1%) women had induction of labor and 64,949 (42.9%) women underwent scheduled caesarean section. Of women who had induction of labor, 10,561 (12.2%) required operative vaginal birth (forceps or vacuum), 20,190 (23.3%) had emergency caesarean section. Severe perineal trauma occurred in 2047 (2.5%) and shoulder dystocia in 3288 (3.8%) of those that had induction of labor and birthed vaginally. Rates of severe adverse maternal outcome were higher in the induction of labor group compared to the scheduled caesarean section cohort (17.2% vs. 12.0%). Higher rates of severe neonatal neurological morbidity (1.1% vs. 0.4%) and other severe neonatal morbidity (12.0% vs. 7.6%) occurred in the induction of labor group compared to the scheduled caesarean section group.

Table 1 details the demographic, obstetric and neonatal differences between induction of labor and scheduled caesarean section cohorts. Women in the scheduled caesarean cohort were older and overweight or obese and had higher rates of previous caesarean section. The induction of labor group had higher proportions of nulliparous women, hypertension and pre-eclampsia, diabetes mellitus and low socioeconomic status.

**Table 1:** Characteristics of study population.

	Total	Induction of labor	Scheduled cesarean	P value
	N = 151,464	N = 86,515	N = 64,949	
**Maternal health and demographic characteristics**				
Maternal age (years)	30 ± 5	29 ± 6	32 ± 5	<0.001
Mother's country of birth				<0.001
Australia, New Zealand, USA, Canada, & Europe	131,780 (87.0%)	75,590 (87.4%)	56,190 (86.5%)	
Indigenous Australian	7858 (5.2%)	4970 (5.7%)	2888 (4.4%)	
Asia and Oceania^a^	8292 (5.5%)	4221 (4.9%)	4071 (6.3%)	
Other^b^	3534 (2.3%)	1734 (2.0%)	1800 (2.8%)	
Maternal BMI (kg/m^2^)				<0.001
Normal (19–24)	38,754 (25.6%)	22,298 (25.8%)	16,456 (25.3%)	
Overweight (25–29)	29,301 (19.3%)	16,259 (18.8%)	13,042 (20.1%)	
Obese (≥30)	35,702 (23.6%)	19,762 (22.8%)	15,940 (24.5%)	
Low socioeconomic status^c^	27,612 (18.4%)	16,932 (19.7%)	10,680 (16.6%)	<0.001
Diabetes mellitus in pregnancy	23,279 (15.4%)	13,983 (16.2%)	9296 (14.3%)	<0.001
Hypertension in pregnancy	9811 (6.5%)	7272 (8.4%)	2539 (3.9%)	<0.001
Pre-eclampsia	4127 (2.7%)	3476 (4.0%)	651 (1.0%)	<0.001
Anemia	9182 (6.1%)	5698 (6.6%)	3484 (5.4%)	<0.001
Drug use	493 (0.3%)	327 (0.4%)	166 (0.3%)	<0.001
Alcohol use	136 (0.1%)	94 (0.1%)	42 (0.1%)	0.005
Smoking	9991 (8.3%)	6301 (9.3%)	3690 (7.0%)	<0.001
**Obstetric history and labor outcomes**				
Nulliparous	47,437 (31.3%)	35,062 (40.5%)	12,375 (19.1%)	<0.001
Assisted conception	8454 (5.6%)	3801 (4.4%)	4653 (7.2%)	<0.001
Previous stillbirth	2440 (1.6%)	1303 (1.5%)	1137 (1.8%)	<0.001
Previous CS	46,328 (30.7%)	2845 (3.3%)	43,483 (67.1%)	<0.001
Chorioamnionitis	185 (0.1%)	166 (0.2%)	19 (0.0%)	<0.001
Antepartum hemorrhage	3604 (2.4%)	1839 (2.1%)	1765 (2.7%)	<0.001
Prolonged second stage labor	2309 (1.5%)	2309 (2.7%)	0 (0.0%)	
Presentation at delivery				<0.001
Cephalic	140,036 (92.5%)	83,963 (97.1%)	56,073 (86.3%)	
Breech	6695 (4.4%)	265 (0.3%)	6430 (9.9%)	
Other^d^	4709 (3.2%)	2287 (2.6%)	2422 (3.9%)	
Method of birth				
Vaginal	55,752 (36.8%)	55,752 (64.4%)	0 (0.0%)	
Operative vaginal birth	10,561 (7.0%)	10,561 (12.2%)	0 (0.0%)	
Planned CS	64,949 (42.9%)	0 (0.0%)	64,949 (100.0%)	
Emergency CS	20,190 (13.3%)	20,190 (23.3%)	0 (0.0%)	
CS for FTP	10,820 (7.1%)	10,820 (12.5%)	0 (0.0%)	
Operative vaginal birth for FTP	4657 (3.1%)	4657 (5.4%)	0 (0.0%)	
CS for NRFS	3972 (2.6%)	3972 (4.6%)	0 (0.0%)	
Operative vaginal birth for NRFS	3654 (2.4%)	3654 (4.2%)	0 (0.0%)	
**Neonatal outcomes**				
Gestational age at birth in completed weeks	39 (38, 40)	39 (38, 40)	38 (38, 39)	<0.001
Birthweight (g)	3960 (3770, 4180)	4018 (3820, 4230)	3875 (3710, 4100)	<0.001
Infant sex				0.006
Male	78,937 (52.1%)	44,822 (51.8%)	34,115 (52.5%)	
Female	72,527 (47.9%)	41,693 (48.2%)	30,834 (47.5%)	
HIE	64 (0.0%)	57 (0.1%)	7 (0.0%)	<0.001
Seizures	130 (0.1%)	81 (0.1%)	49 (0.1%)	0.23
Sepsis	7425 (4.9%)	5972 (6.9%)	1453 (2.2%)	<0.001
NEC	4 (0.0%)	2 (0.0%)	2 (0.0%)	0.77
Birth trauma	1354 (0.9%)	1192 (1.4%)	162 (0.2%)	<0.001
Hypoglycemia	7740 (5.1%)	4472 (5.2%)	3268 (5.0%)	0.23
NICU admission	1393 (0.9%)	785 (0.9%)	608 (0.9%)	0.57
SCN admission	21,098 (14.0%)	12,705 (14.7%)	8393 (12.9%)	<0.001

Data are presented as mean ± SD or median (IQR) for continuous measures, and N (%) for categorical measures.

USA, United States of America; BMI, Body Mass Index, CS, Cesarean Section; FTP, Failure to Progress; NRFS, Non-Reassuring Fetal Status; IOL, Induction of labor; HIE, Hypoxic Ischemic Encephalopathy; NEC, Necrotizing Enterocolitis; NICU, Neonatal Intensive Care Unit; SCN, Special Care Nursery.

a Excluding Australia and New Zealand.

b Africa, Latin America, and Caribbean.

c Lowest Quintile of SEIFA score.

d Abnormal Lie/Compound/Hand/Cephalic malposition.

Table 2 presents the multivariable logistic regression analyses for the primary outcomes stratified by method of planned birth and gestational age at delivery. Compared to induction of labor at 38^+0^–38^+6^ weeks (referent category), induction of labor at ≥41^+0^ weeks (aOR 1.28, 95% CI 1.21, 1.35) and scheduled caesarean section at 37^+0^–37^+6^ weeks (aOR 1.18, 95% CI 1.08, 1.28) were associated with greater odds of severe adverse maternal outcome. However, scheduled caesarean section at 39^+0^–39^+6^ weeks (aOR 0.75, 95% CI 0.70, 0.80) was associated with lower odds of this outcome. Neither method of, nor gestational age at planned birth were associated with the odds of perinatal mortality.

**Table 2:** Primary outcomes by method of planned birth and gestational age.

	N (%)	OR (95% CI)	P-value	aOR (95% CI)	P-value
**Severe adverse maternal outcome**				a	
Induction of labor at 37^+0^–37^+6^ weeks	1106 (18.0)	1.12 (1.04, 1.21)	0.002	1.02 (0.95, 1.11)	0.558
**Induction of labor at 38^+0^–38^+6^ weeks**	2986 (16.4)	**Referent**	**Referent**	**Referent**	**Referent**
Induction of labor at 39^+0^–39^+6^ weeks	3121 (16.0)	0.97 (0.92, 1.03)	0.339	0.99 (0.94, 1.05)	0.716
Induction of labor at 40^+0^–40^+6^ weeks	3507 (16.3)	1.00 (0.94, 1.05)	0.879	1.00 (0.95, 1.06)	0.894
Induction of labor at ≥41^+0^ weeks	4115 (19.6)	1.24 (1.18, 1.31)	<0.001	1.28 (1.21, 1.35)	<0.001
Scheduled CS at 37^+0^–37^+6^ weeks	1132 (17.9)	1.11 (1.03, 1.20)	0.005	1.18 (1.08, 1.28)	<0.001
Scheduled CS at 38^+0^–38^+6^ weeks	3554 (12.0)	0.70 (0.66, 0.74)	<0.001	0.85 (0.80, 0.90)	<0.001
Scheduled CS at 39^+0^–39^+6^ weeks	2522 (10.4)	0.59 (0.56, 0.63)	<0.001	0.75 (0.70, 0.80)	<0.001
Scheduled CS at 40^+0^–40^+6^ weeks	445 (12.5)	0.73 (0.66, 0.81)	<0.001	0.82 (0.73, 0.92)	<0.001
Scheduled CS at ≥41^+0^ weeks	167 (14.1)	0.84 (0.71, 0.99)	0.043	0.94 (0.79, 1.12)	0.502
**Perinatal mortality**				b	
Induction of labor at 37^+0^–37^+6^ weeks	1 (0.02)	0.60 (0.07, 5.09)	0.636	0.52 (0.06, 4.54)	0.557
**Induction of labor at 38^+0^–38^+6^ weeks**	5 (0.03)	**Referent**	**Referent**	**Referent**	**Referent**
Induction of labor at 39^+0^–39^+6^ weeks	6 (0.03)	1.12 (0.34, 3.68)	0.848	1.48 (0.46, 4.75)	0.511
Induction of labor at 40^+0^–40^+6^ weeks	6 (0.03)	1.02 (0.31, 3.34)	0.976	1.60 (0.51, 5.02)	0.423
Induction of labor at ≥41^+0^ weeks	7 (0.03)	1.21 (0.39, 3.83)	0.739	2.13 (0.67, 6.78)	0.201
Scheduled CS at 37^+0^–37^+6^ weeks	2 (0.03)	1.15 (0.22, 5.95)	0.864	0.71 (0.14, 3.70)	0.682
Scheduled CS at 38^+0^–38^+6^ weeks	8 (0.03)	0.99 (0.32, 3.02)	0.985	1.04 (0.36, 3.00)	0.949
Scheduled CS at 39^+0^–39^+6^ weeks	2 (0.01)	0.30 (0.06, 1.55)	0.151	0.38 (0.08, 1.87)	0.233
Scheduled CS at 40^+0^–40^+6^ weeks	0 (0)	e	e	e	e
Scheduled CS at ≥41^+0^ weeks	0 (0)	e	e	e	e
**Severe neonatal neurological morbidity**				c	
Induction of labor at 37^+0^–37^+6^ weeks	83 (1.7)	1.17 (0.91, 1.51)	0.218	1.1 (0.86, 1.42)	0.448
**Induction of labor at 38^+0^–38^+6^ weeks**	225 (1.5)	**Referent**	**Referent**	**Referent**	**Referent**
Induction of labor at 39^+0^–39^+6^ weeks	193 (1.1)	0.76 (0.62, 0.92)	0.005	0.84 (0.69, 1.02)	0.081
Induction of labor at 40^+0^–40^+6^ weeks	183 (0.9)	0.64 (0.52, 0.77)	<0.001	0.72 (0.59, 0.89)	0.002
Induction of labor at ≥41^+0^ weeks	221 (1.2)	0.81 (0.67, 0.97)	0.026	0.87 (0.72, 1.06)	0.18
Scheduled CS at 37^+0^–37^+6^ weeks	50 (0.9)	0.64 (0.47, 0.88)	0.005	0.59 (0.43, 0.81)	0.001
Scheduled CS at 38^+0^–38^+6^ weeks	107 (0.4)	0.27 (0.21, 0.33)	<0.001	0.28 (0.22, 0.36)	<0.001
Scheduled CS at 39^+0^–39^+6^ weeks	81 (0.4)	0.24 (0.19, 0.31)	<0.001	0.26 (0.2, 0.33)	<0.001
Scheduled CS at 40^+0^–40^+6^ weeks	16 (0.5)	0.32 (0.19, 0.54)	<0.001	0.32 (0.19, 0.54)	<0.001
Scheduled CS at ≥41^+0^ weeks	5 (0.4)	0.30 (0.13, 0.74)	0.009	0.31 (0.13, 0.75)	0.009
**Other severe neonatal morbidity**				d	
Induction of labor at 37^+0^–37^+6^ weeks	1265 (20.9)	1.43 (1.33, 1.54)	<0.001	1.35 (1.24, 1.46)	<0.001
**Induction of labor at 38^+0^–38^+6^ weeks**	2809 (15.6)	**Referent**	**Referent**	**Referent**	**Referent**
Induction of labor at 39^+0^–39^+6^ weeks	2089 (10.8)	0.66 (0.62, 0.7)	<0.001	0.79 (0.75, 0.85)	<0.001
Induction of labor at 40^+0^–40^+6^ weeks	1892 (8.9)	0.53 (0.5, 0.56)	<0.001	0.69 (0.64, 0.73)	<0.001
Induction of labor at ≥41^+0^ weeks	2327 (11.2)	0.68 (0.64, 0.72)	<0.001	0.93 (0.87, 0.99)	0.032
Scheduled CS at 37^+0^–37^+6^ weeks	1033 (16.4)	1.07 (0.99, 1.15)	0.104	0.86 (0.78, 0.94)	0.001
Scheduled CS at 38^+0^–38^+6^ weeks	2165 (7.4)	0.43 (0.41, 0.46)	<0.001	0.43 (0.4, 0.46)	<0.001
Scheduled CS at 39^+0^–39^+6^ weeks	1457 (6.0)	0.35 (0.32, 0.37)	<0.001	0.38 (0.35, 0.41)	<0.001
Scheduled CS at 40^+0^–40^+6^ weeks	181 (5.1)	0.29 (0.25, 0.34)	<0.001	0.31 (0.26, 0.36)	<0.001
Scheduled CS at ≥41^+0^ weeks	69 (5.9)	0.34 (0.26, 0.43)	<0.001	0.38 (0.29, 0.48)	<0.001

Data are presented as adjusted odds ratio (aOR) 95% confidence interval (CI).

a Adjusted for maternal age, maternal country of birth, body mass index (BMI), low socioeconomic status (SES), assisted reproductive technology (ART), previous stillbirth, previous cesarean section (CS), presentation at birth, illicit drugs, parity, antepartum hemorrhage, diabetes, hypertension, and birthweight centile.

b Adjusted for BMI, prolonged second stage labor (>3 h), antepartum hemorrhage, and diabetes.

c Adjusted for maternal age, maternal country of birth, BMI, low SES, smoking, illicit drugs, parity, antepartum hemorrhage, diabetes, prolonged second stage labor, presentation at birth, infant sex, and birthweight centile.

d Adjusted for maternal age, maternal country of birth, BMI, assisted conception, previous stillbirth, previous CS, presentation at birth, prolonged second stage labor, illicit drugs, parity, antepartum hemorrhage, diabetes, hypertension, birthweight centile, and infant sex.

e No observations.

The odds of severe neonatal neurological morbidity were significantly lower for induction of labor at 40^+0^–40^+6^ weeks (aOR 0.72, 95% CI 0.59, 0.89), scheduled caesarean section at 37^+0^–37^+6^ weeks (aOR 0.59, 95% CI 0.43, 0.81), at 39^+0^–39^+6^ weeks (aOR 0.26, 95% CI 0.2, 0.33), and at ≥41^+0^ weeks (aOR 0.31, 95% CI 0.13, 0.75) compared to induction of labor at 38^+0^–38^+6^ weeks. The odds of other severe neonatal morbidity were highest after induction of labor at 37^+0^–37^+6^ weeks (aOR 1.35, 95% CI 1.24, 1.46), and lowest in those following scheduled caesarean section at 40^+0^–40^+6^ weeks (aOR 0.31, 95% CI 0.26, 0.36).

**Fig. 2:**
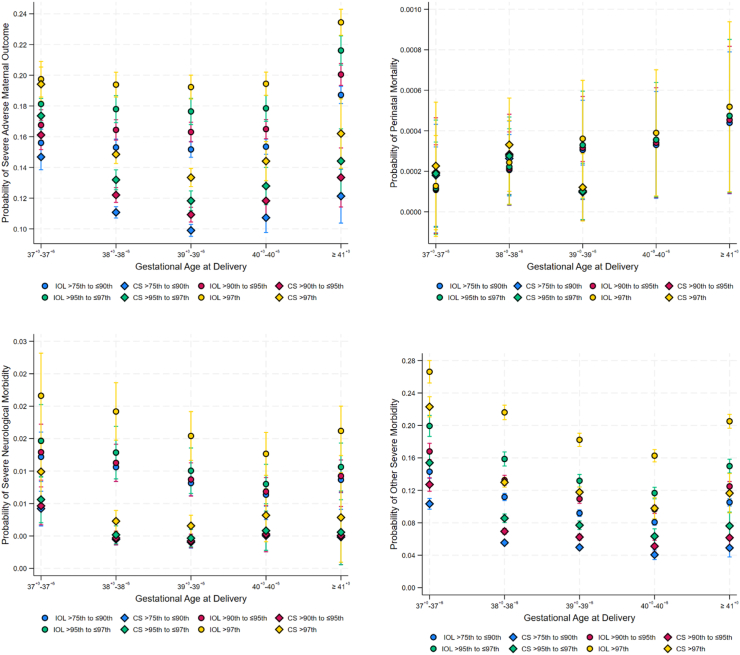
The probabilities and 95% confidence intervals (CI) of study outcomes according to gestational age at delivery, method of planned birth, and birthweight centile. IOL, Induction of Labor; CS, Scheduled Caesarean Section.

The marginal probabilities of severe adverse maternal outcomes, severe neurological and other severe neonatal morbidity were all higher in the induction of labor cohort compared to scheduled caesarean section across all gestational age categories (Fig. 2). Regardless of method of planned birth the marginal probabilities of severe adverse maternal outcome were highest in the birthweight >97th centile group and lowest in the ≥75th–≤90th category. The marginal probabilities of severe neurological and other severe neonatal morbidity were highest in the birthweight >97th centile group following induction of labor at 37^+0^–37^+6^ weeks (Fig. 2).

The secondary outcomes only pertain to women undergoing induction of labor. Of this cohort, the odds of operative vaginal birth were significantly lower at 37^+0^–37^+6^ weeks (aOR 0.82, 95% CI 0.74, 0.92). Induction of labor at 39^+0^–39^+6^ weeks was associated with the lowest odds of emergency caesarean section (aOR 0.88, 95% CI 0.80, 0.97). The odds of emergency operative birth for failure to progress, prolonged second stage of labor >3 h and severe perineal trauma were all significantly higher after induction of labor at 40^+0^–40^+6^ weeks and ≥41^+0^ weeks respectively. The odds of shoulder dystocia were lowest at 37^+0^–37^+6^ weeks (aOR 0.78, 95% CI 0.66, 0.93) but increased when induction of labor was performed after 39^+0^ weeks (Table 3).

**Table 3:** Secondary outcomes.

	N (%)	OR (95% CI)	P-value	aOR (95% CI)	P-value
**Operative vaginal birth**				a	
37^+0^–37^+6^ weeks	634 (13.1)	0.97 (0.88, 1.06)	0.486	0.82 (0.74, 0.92)	<0.001
**38^+0^–38^+6^ weeks**	1960 (13.5)	**Referent**	**Referent**	**Referent**	**Referent**
39^+0^–39^+6^ weeks	2404 (15.5)	1.18 (1.11, 1.26)	<0.001	1.02 (0.95, 1.10)	0.555
40^+0^–40^+6^ weeks	2854 (17.4)	1.36 (1.27, 1.44)	<0.001	1.02 (0.95, 1.09)	0.664
≥41^+0^ weeks	2709 (18.0)	1.42 (1.33, 1.51)	<0.001	0.97 (0.9, 1.04)	0.356
**Emergency cesarean section**				b	
37^+0^–37^+6^ weeks	1287 (23.4)	1.04 (0.97, 1.12)	0.318	0.92 (0.80, 1.05)	0.218
**38^+0^–38^+6^ weeks**	3706 (22.7)	**Referent**	**Referent**	**Referent**	**Referent**
39^+0^–39^+6^ weeks	4016 (23.5)	1.04 (0.99, 1.10)	0.114	0.88 (0.80, 0.97)	0.011
40^+0^–40^+6^ weeks	5140 (27.5)	1.29 (1.23, 1.36)	<0.001	1.00 (0.91, 1.10)	0.974
≥41^+0^ weeks	6041 (32.9)	1.67 (1.59, 1.75)	<0.001	1.07 (0.98, 1.18)	0.143
**Emergency operative birth for failure to progress**				c	
37^+0^–37^+6^ weeks	806 (13.1)	0.90 (0.83, 0.98)	0.017	0.82 (0.74, 0.91)	<0.001
**38^+0^–38^+6^ weeks**	2621 (14.4)	**Referent**	**Referent**	**Referent**	**Referent**
39^+0^–39^+6^ weeks	3135 (16.1)	1.14 (1.08, 1.21)	<0.001	1.05 (0.98, 1.12)	0.185
40^+0^–40^+6^ weeks	4128 (19.2)	1.42 (1.34, 1.49)	<0.001	1.14 (1.07, 1.22)	<0.001
≥41^+0^ weeks	4787 (22.7)	1.76 (1.67, 1.85)	<0.001	1.19 (1.12, 1.27)	<0.001
**Prolonged second stage of labor > 3 h**				d	
37^+0^–37^+6^ weeks	1077 (17.5)	1.06 (0.98, 1.14)	0.138	0.93 (0.86, 1.02)	0.112
**38^+0^–38^+6^ weeks**	3055 (16.7)	**Referent**	**Referent**	**Referent**	**Referent**
39^+0^–39^+6^ weeks	3453 (17.7)	1.07 (1.01, 1.13)	0.014	1.05 (0.99, 1.12)	0.087
40^+0^–40^+6^ weeks	4399 (20.4)	1.28 (1.22, 1.35)	<0.001	1.17 (1.11, 1.25)	<0.001
≥41^+0^ weeks	5384 (25.6)	1.71 (1.63, 1.8)	<0.001	1.53 (1.45, 1.63)	<0.001
**Shoulder dystocia**				e	
37^+0^–37^+6^ weeks	171 (2.8)	0.82 (0.69, 0.97)	0.02	0.78 (0.66, 0.93)	0.005
**38^+0^–38^+6^ weeks**	620 (3.4)	**Referent**	**Referent**	**Referent**	**Referent**
39^+0^–39^+6^ weeks	679 (3.5)	1.03 (0.92, 1.15)	0.657	1.17 (1.05, 1.31)	0.006
40^+0^–40^+6^ weeks	832 (3.9)	1.14 (1.03, 1.27)	0.013	1.40 (1.25, 1.56)	<0.001
≥41^+0^ weeks	986 (4.7)	1.40 (1.26, 1.55)	<0.001	1.86 (1.67, 2.08)	<0.001
**Severe perineal trauma**				f	
37^+0^–37^+6^ weeks	106 (1.8)	0.98 (0.79, 1.22)	0.864	0.88 (0.71, 1.11)	0.277
**38^+0^–38^+6^ weeks**	320 (1.8)	**Referent**	**Referent**	**Referent**	**Referent**
39^+0^–39^+6^ weeks	396 (2.2)	1.18 (1.01, 1.37)	0.031	1.14 (0.98, 1.33)	0.081
40^+0^–40^+6^ weeks	503 (2.5)	1.40 (1.21, 1.61)	<0.001	1.28 (1.11, 1.48)	0.001
≥41^+0^ weeks	722 (3.7)	2.05 (1.8, 2.34)	<0.001	1.89 (1.65, 2.17)	<0.001

Data are presented as adjusted odds ratio (aOR) 95% confidence interval (CI).

a Adjusted for maternal age, maternal country of birth, body mass index (BMI), low socioeconomic status (SES), smoking, assisted reproductive technology (ART), previous cesarean section (CS), prolonged second stage labor (>3 h), parity, diabetes, birthweight centile, and infant sex.

b Adjusted for maternal age, maternal country of birth, body mass index (BMI), smoking, ART, previous stillbirth, previous CS, prolonged second stage labor, parity, hypertension, birthweight centile, and infant sex.

c Adjusted for maternal age, maternal country of birth, previous stillbirth, previous CS, presentation at birth, prolonged second stage labor, parity, antepartum hemorrhage, diabetes, infant sex, and birthweight centile.

d Adjusted for maternal age, maternal country of birth, BMI, smoking, previous CS, presentation at birth, alcohol, parity, antepartum hemorrhage, diabetes, hypertension, birthweight centile, and infant sex.

e Adjusted for maternal age, maternal country of birth, low SES, prolonged second stage labor, presentation at birth, illicit drugs, diabetes, birthweight centile, and infant sex.

f Adjusted for maternal age, maternal country of birth, previous CS, prolonged second stage labor, parity, hypertension, birthweight centile, and infant sex.

**Fig. 3:**
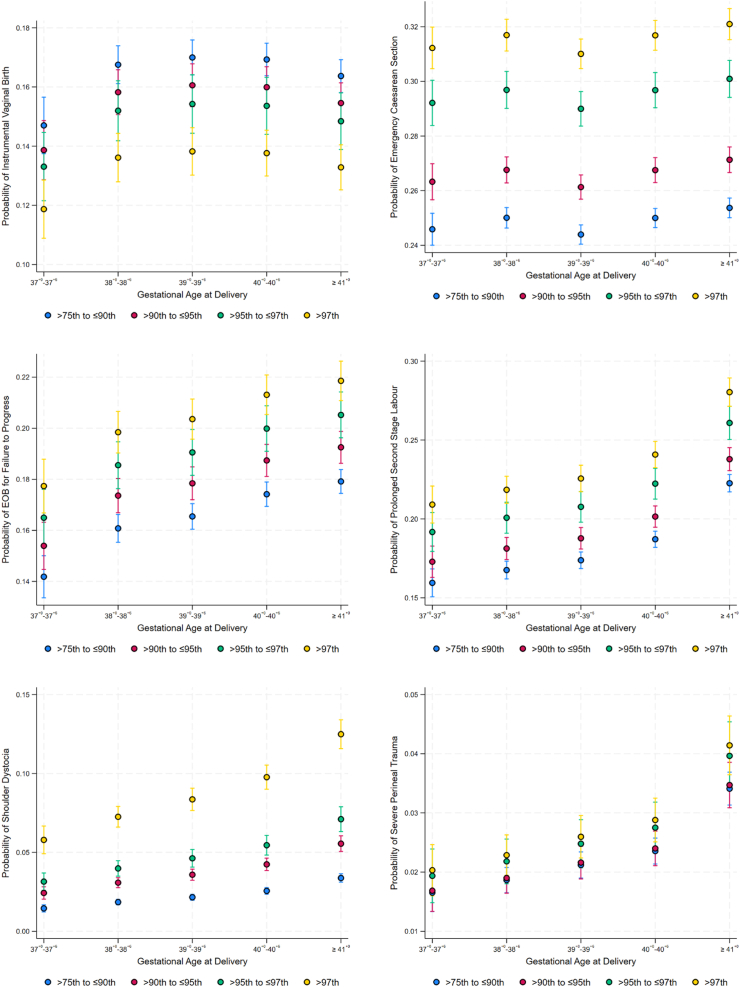
The probabilities and 95% confidence intervals (CI) of adverse intrapartum outcomes in women following induction of labor by gestational age at delivery and birthweight centile. EOB, Emergency Operative Birth.

Fig. 3 depicts the marginal probabilities of adverse intrapartum outcomes in women following induction of labor stratified by gestational age category and birthweight centile. Across all birthweight centiles (>75th–≤90th centiles, >90th–≤95th centiles, >95th–≤97th centiles, and >97th centiles), the probability of operative vaginal birth increased with gestational age from 37^+0^ to 39^+6^ weeks but then decreased thereafter. The probability of emergency caesarean section was lowest at 39^+0^–39^+6^ weeks. The probabilities for emergency operative birth for failure to progress, prolonged second stage of labor, shoulder dystocia and perineal trauma increased with gestational age and were highest for infants with birthweight >97th centile.

## Discussion

The key findings of this large study of term infants with birthweight >75th centile for gestational age, are, firstly, compared to induction of labor at 38^+0^–38^+6^ weeks, scheduled caesarean section at 39^+0^–39^+6^ weeks was associated with the lowest odds of severe adverse maternal outcomes, severe neonatal neurological morbidity and other severe neonatal morbidity. Secondly, although planned birth at 39^+0^–39^+6^ weeks was associated with the lowest marginal probabilities of severe maternal outcome, perinatal mortality, severe neonatal neurological morbidity and other severe neonatal morbidity, the lowest probabilities were seen in the scheduled caesarean cohort regardless of birthweight centile category. For women that underwent induction of labor, the odds of emergency caesarean section were lowest at 39^+0^–39^+6^ weeks whilst the odds of emergency operative birth for failure to progress, prolonged second stage of labor and perineal trauma were greater after 40^+0^ weeks' gestation. Collectively, our results suggest that planned birth at 39^+0^–39^+6^ weeks is associated with better maternal and infant outcomes compared to induction of labor at 38^+0^–38^+6^ weeks for infants with birthweight >75th centile. Our results also suggest that scheduled caesarean section may be associated with better outcomes compared to induction of labor.

Although our results are similar to that of Boulvain et al., ^27^ who showed that induction of labor at 37^+0^–38^+6^ weeks for women with large for gestational age fetuses, resulted in a reduced risk of shoulder dystocia associated complications, we provide additional information demonstrating that induction beyond this period was associated with increased odds of shoulder dystocia and severe perineal trauma. Our results are also broadly consistent with the recent Big Baby trial which showed that women with a large for gestational age infant (defined as estimated fetal weight >90th centile) had a lower relative risk of adverse outcomes including shoulder dystocia and emergency caesarean section following induction of labor at 38^+0^–38^+4^ weeks compared to those having standard care. In a systematic review and meta-analysis by Badr et al., ^28^ the authors reported that induction of labor for infants with suspected macrosomia (defined as estimated fetal weight ≥90th centile for gestation) before 40^+0^ weeks gestation was associated with reduced odds of shoulder dystocia without an increase in other adverse outcomes, such as emergency caesarean section or admission to the neonatal intensive care unit. Our results suggest that even for infants below this threshold, planned birth results in better maternal and perinatal outcomes. In another retrospective cohort study of expectant management at term,^29^ the rate of severe neonatal morbidity was for infants with birthweight >90th centile was lowest at 39^+0^–39^+6^ weeks. A key distinction between our study and that of others is our evaluation of method of planned birth on maternal and perinatal outcomes. Much of the published literature focusses on outcomes following planned birth by induction of labor. Our results therefore are pertinent to the many women with a potentially larger infant (estimated fetal weight > 75th–90th centile), who opt for a scheduled caesarean section because of concerns of complications during vaginal birth. Our results are also consistent with recent evidence^30^ showing that in low-risk women with infants with birthweight >90th centile, scheduled caesarean section at 39^+0^–39^+6^ weeks compared to induction of labor, was associated with 71% lower odds of severe neonatal neurological morbidity and 25% lower odds severe maternal outcome.

In another study by Baudry et al., ^31^ induction of labor at 39 weeks of gestation for large for gestational age infants (defined as estimated fetal weight ≥95th centile for gestation), was associated with significantly lower rates of neonatal acidosis compared to expectant management (5.2% vs. 16.9%). In contrast to our results, Corbett et al., ^32^ found no significant difference in major maternal morbidity and adverse neonatal outcomes between planned birth (induction of labor or scheduled caesarean section) at 38^+0^ and 40^+6^ weeks' gestation compared to expectant management for infants with estimated fetal weight >4000 g. In Corbett et al.'s study, induction of labor in nulliparous women was associated with increased risk of anal sphincter injury and postpartum hemorrhage when compared to expectant management. The 2023 Cochrane systematic review^15^ on induction of labor at or near term for suspected fetal macrosomia (birthweight > 4000 g) found that compared to expectant management, induction of labor was associated with lower risk of infant fractures (risk ratio (RR) 0.20, 95% CI 0.05–0.79) and shoulder dystocia (RR 0.60, 95% CI 0.37–0.98) but no clear effect on caesarean section, instrumental vaginal birth brachial plexus injury, low 5 min Apgar score or card artery pH. However, this review, unlike our current study, did not evaluate the effect of gestational age at birth or the method of planned birth on other serious perinatal outcomes. Nevertheless, the authors concluded that although induction of labor between 38^+0^–38^+6^ weeks was likely to minimize the risks of iatrogenic prematurity, it may not have much impact on birthweight or reduction in birth trauma. Our study provides additional detail of differences in outcomes both by gestational age and method of planned birth and thus may help clinicians and parents jointly decide the most appropriate mode and timing of birth. Although the evidence^30^^,^^33^, ^34^, ^35^ is now clear that induction of labor compared with expectant management is not associated with an increased risk of caesarean section, there are additional considerations for women with large infants who undergo induction of labor. Moldéus et al.^36^ and Combs et al.^37^ found that rates of emergency caesarean delivery were higher in women undergoing induction of labor compared to those managed expectantly.

Our study has several strengths. A major strength of our study is our inclusion of real-world data of women undergoing induction of labor and those having scheduled caesarean section which allowed us to evaluate the individual impact of both interventions on our study outcomes. To our knowledge this is one of the largest cohorts of women with large infants analyzed in this manner. We evaluate outcomes by method of planned birth and gestational age stratified for infant birthweight. Our study population included women from diverse ethnic and socioeconomic backgrounds increasing potential generalizability of our findings. Our dataset had very low levels of missing data. Furthermore, we selected confounders and effect measure modifiers that were clinically relevant, thus minimizing bias from inappropriate adjustment. We adjusted for clinically relevant confounders and considered potential associations with changes in practice and stratified them by effect measure modifiers. We however did not adjust for multiple comparisons, which could increase the risk of false positive results. Finally, our choice of components for our composite outcomes was guided by recent publications^21^^,^^38^ defining core outcomes (applicable regardless of gestational age, birth weight, illness severity, infant population or clinical setting) identified as important by key international stakeholders.

A key limitation of our analyses is our use of birthweight rather than estimated fetal weight. We acknowledge that it is self-evident that decisions regarding timing of birth or other aspects of pregnancy management require information that is available before birth. However, as universal late-pregnancy ultrasound is not routine in Australia (or worldwide), for most pregnant women, prenatal assessment of fetal size is first made clinically (by palpation of symphysis fundal height measurement), notwithstanding its obvious constraints before objective measurement using ultrasound. The main objective of our study was to illustrate the potential for mitigating adverse outcomes for large infants by selection of the optimum method and gestation of planned birth (induction of labor or scheduled caesarean section) stratified by infant size using birthweight as a surrogate. Other limitations of this study include our inability to account for the effects of unmeasured confounding or selection bias. We could not account for the potential impact of changes in clinical practice over the study period. We were also not able to completely discount the possibility of other undocumented indications which may have influenced outcomes. The use of any retrospective dataset is potentially limited by temporal variations or errors in coding, all of which could potentially have influenced data quality and stability over the study period. We assumed that gestational age was correctly ascertained at the individual patient level, accurately recorded and reported, albeit we accept the inherent limitations of this assumption. We confirmed the veracity of data by multiple means including repeated cycles of screening, identifying and subsequent editing of unclear data. We were also not able to demonstrate any significant difference in perinatal mortality between both cohorts and each gestational week due to the low number of observations in the dataset. Finally, we were not able to correlate our findings with long term maternal or offspring outcomes as this information was not available in the dataset. This is particularly pertinent as caesarean section is associated with increased future risks of placenta accreta spectrum complications, uterine rupture and other consequences.

In conclusion, when deciding the optimum mode and timing of birth for women with a large infant women and healthcare providers must balance the risks of prolonged labor, emergency operative birth and adverse neonatal and maternal outcomes against the likelihood of successful vaginal birth. The results from our study may thus help to contextualize some of the intrapartum, maternal and perinatal risks both by gestational age and method of planned birth for consumers and healthcare professionals when deciding timing and method of birth for women with large infants.

Our findings suggest that planned birth between 39^+0^–39^+6^ weeks is associated with lower odds for a variety of adverse outcomes. Our results also demonstrate that at equivalent gestations, scheduled caesarean section, compared to induction of labor is associated with lower odds adverse maternal and neonatal outcomes. The greatest risks of severe maternal and neonatal outcomes occur in infants with birthweight >97th centile for gestational age regardless of method or gestation of planned birth.

## Contributors

SK conceived the study, provided overall supervision, reviewed, and extensively revised all versions of the manuscript. KC compiled the database and analyzed the data from the Queensland perinatal dataset with input from all authors. GS, KC, JH, and SK wrote the first draft and verified the underlying data. All authors had access to all data in the study, contributed to writing of the manuscript, and had final responsibility to submit for publication. All authors read and approved the final version of the manuscript.

## Data sharing statement

All code, scripts, and data used to produce the results in this article will be available to any researcher provided appropriate ethics approval, inter-institutional data sharing agreements and other regulatory requirements are in place. Additional specific approval from the Data Custodian of the Statistical Services Branch of Queensland's Department of Health will also be required. Some approvals are outside the remit of the authors.

## Declaration of interests

No conflicts of interests are reported.

## References

[1] Culliney K.A.T., Parry G.K., Brown J., Crowther C.A. (2016). Regimens of fetal surveillance of suspected large-for-gestational-age fetuses for improving health outcomes. Cochrane Database Syst Rev.

[2] Royal Australian, New Zealand College of Obstetricians & Gynaecologists (2021). Clinical Guidance Statement: Diagnosis and Management of Suspected Fetal Macrosomia.

[3] Joseph F.A., Hyett J.A., Schluter P.J. (2020). New Australian birthweight centiles. Med J Aust.

[4] American College of Obstetricians & Gynecologists (2020). Macrosomia: ACOG practice bulletin summary, number 216. Obstet Gynecol.

[5] Lahmann P.H., Wills R.A., Coory M. (2009). Trends in birth size and macrosomia in Queensland, Australia, from 1988 to 2005. Paediatr Perinat Epidemiol.

[6] Henriksen T. (2008). The macrosomic fetus: a challenge in current obstetrics. Acta Obstet Gynecol Scand.

[7] Koyanagi A., Zhang J., Dagvadorj A. (2013). Macrosomia in 23 developing countries: an analysis of a multicountry, facility-based, cross-sectional survey. Lancet.

[8] AIHW (2023). Australia's mothers and babies. https://www.aihw.gov.au/reports/mothers-babies/australias-mothers-babies/contents/baby-outcomes/birthweight.

[9] Ye W., Luo C., Huang J., Li C., Liu Z., Liu F. (2022). Gestational diabetes mellitus and adverse pregnancy outcomes: systematic review and meta-analysis. BMJ.

[10] Murphy H.R., Howgate C., O'Keefe J. (2021). Characteristics and outcomes of pregnant women with type 1 or type 2 diabetes: a 5-year national population-based cohort study. Lancet Diabetes Endocrinol.

[11] Hare M.J.L., Barzi F., Boyle J.A. (2020). Diabetes during pregnancy and birthweight trends among Aboriginal and non-Aboriginal people in the Northern Territory of Australia over 30 years. Lancet Reg Health West Pac.

[12] Triggs T., Crawford K., Hong J., Clifton V., Kumar S. (2024). The influence of birthweight on mortality and severe neonatal morbidity in late preterm and term infants: an Australian cohort study. Lancet Reg Health West Pac.

[13] Scifres C.M. (2021). Short- and long-term outcomes associated with large for gestational age birth weight. Obstet Gynecol Clin North Am.

[14] Bukowski R., Hansen N.I., Willinger M. (2014). Fetal growth and risk of stillbirth: a population-based case-control study. PLoS Med.

[15] Boulvain M., Thornton J.G. (2023). Induction of labour at or near term for suspected fetal macrosomia. Cochrane Database Syst Rev.

[16] National Institute for Health and Care Excellence (2021). Inducing labour. NICE guideline [NG207]. https://www.nice.org.uk/guidance/ng207.

[17] Dobbins T.A., Sullivan E.A., Roberts C.L., Simpson J.M. (2012). Australian national birthweight percentiles by sex and gestational age, 1998–2007. Med J Aust.

[18] Queensland Health (2016). https://www.health.qld.gov.au/hsu/collections/pdc.

[19] (2021). Induction of Labour for Suspected Fetal Macrosomia: Inducing labour: Evidence Review A.

[20] (2020). Macrosomia: ACOG practice bulletin, number 216. Obstet Gynecol.

[21] Webbe J.W.H., Duffy J.M.N., Afonso E. (2020). Core outcomes in neonatology: development of a core outcome set for neonatal research. Arch Dis Child Fetal Neonatal Ed.

[22] Wolff R.F., Moons K.G.M., Riley R.D. (2019). PROBAST: a tool to assess the risk of bias and applicability of prediction model studies. Ann Intern Med.

[23] Heinze G., Wallisch C., Dunkler D. (2018). Variable selection - a review and recommendations for the practicing statistician. Biom J.

[24] Australian Bureau of Statistics (2011). https://www.abs.gov.au/ausstats/abs@.nsf/DetailsPage/2033.0.55.0012011.

[25] Medeiros R. (2016). Handling Missing Data in Stata: Imputation and Likelihood-based Approaches.

[26] von Elm E., Altman D.G., Egger M. (2007). The Strengthening the Reporting of Observational Studies in Epidemiology (STROBE) statement: guidelines for reporting observational studies. Ann Intern Med.

[27] Boulvain M., Senat M.V., Perrotin F. (2015). Induction of labour versus expectant management for large-for-date fetuses: a randomised controlled trial. Lancet.

[28] Badr D.A., Carlin A., Kadji C., Kang X., Cannie M.M., Jani J.C. (2024). Timing of induction of labor in suspected macrosomia: retrospective cohort study, systematic review and meta-analysis. Ultrasound Obstet Gynecol.

[29] Hong J., Crawford K., Odibo A.O., Kumar S. (2023). Risks of stillbirth, neonatal mortality, and severe neonatal morbidity by birthweight centiles associated with expectant management at term. Am J Obstet Gynecol.

[30] Crawford K., Carlo W.A., Odibo A., Papageorghiou A., Tarnow-Mordi W., Kumar S. (2025). Perinatal mortality and other severe adverse outcomes following planned birth at 39 weeks versus expectant management in low-risk women: a population based cohort study. EClinicalMedicine.

[31] Baudry M., Eyraud J.L., Aubard Y., Bru N., Coste Mazeau P. (2022). Impact of induction of labor in fetal macrosomia: comparative series from 256 cases. Arch Gynecol Obstet.

[32] Corbett G.A., Hunter S., Javaid A. (2023). Non-diabetic fetal macrosomia: outcomes of elective delivery versus expectant management. Ir J Med Sci.

[33] Grobman W.A., Rice M.M., Reddy U.M. (2018). Labor induction versus expectant management in low-risk nulliparous women. N Engl J Med.

[34] Grobman W.A., Caughey A.B. (2019). Elective induction of labor at 39 weeks compared with expectant management: a meta-analysis of cohort studies. Am J Obstet Gynecol.

[35] Hong J., Atkinson J., Roddy Mitchell A. (2023). Comparison of maternal labor-related complications and neonatal outcomes following elective induction of labor at 39 Weeks of gestation vs expectant management: a systematic review and meta-analysis. JAMA Netw Open.

[36] Moldéus K., Cheng Y.W., Wikström A.K., Stephansson O. (2017). Induction of labor versus expectant management of large-for-gestational-age infants in nulliparous women. PLoS One.

[37] Combs C.A., Singh N.B., Khoury J.C. (1993). Elective induction versus spontaneous labor after sonographic diagnosis of fetal macrosomia. Obstet Gynecol.

[38] Lee S.I., Hanley S., Vowles Z. (2023). The development of a core outcome set for studies of pregnant women with multimorbidity. BMC Med.

