# Supplementary material for: Optimal timing and mode of planned birth for term, large infants: a retrospective, population-based cohort study
Source: eClinicalMedicine. 2025 Jul 17;86:103366. doi: 10.1016/j.eclinm.2025.103366 (PMC12302999; doi:10.1016/j.eclinm.2025.103366)
Supplement: Supplementary Table S1 [file mmc1.docx]

**Supplementary Table 1** ICD-10 codes relevant to maternal characteristics and neonatal outcomes.

| **Maternal Health and Obstetric History** | **ICD-10 Codes** |
| --- | --- |
| Diabetes Mellitus | E1011, E1015, E1021, E1022, E1023, E1029, E1031, E1033, E1034, E1036, E1040, E1041, E1042, E1043, E1049, E1051, E1061, E1064, E1065, E1069, E1071, E1073, E108, E109, E1101, E1111, E1121, E1122, E1131, E1134, E1136, E1140, E1142, E1143, E1153, E1161, E1162, E1164, E1165, E1171, E1172, E1173, E118, E119, E1322, E1329, E1331, E1342, E1364, E1365, E1371, E1372, E138, E139, E1439, E1443, E1464, E149, G590, O240, O241, O2411, O2412, O2413, O2414, O2419, O242, O2422, O2423, O2424, O2429, O243, O2431, O2432, O2433, O2434, O2439, O244, O2441, O2442, O2443, O2444, O2449, O2453, O2459, O249, O2492, O2493, O2499, O2508, O2509, P700, P701 |
| Hypertension | I10, I110, I119, I120, I129, I130, I131, I139, I150, I151, I152, I158, I159, I272, O10, O100, O102, O103, O104, O109, R030, O13, O16, O120, P000 |
| Preeclampsia | O120, O121, O122, O13, O140, O141, O142, O149, O150, O151, O159, O16, P000 |
| Antepartum Haemorrhage | O458, O459, O460, O468, O469, P021 |
| **Neonatal Morbidity** | **ICD-10 Codes** |
| Birth Asphyxia | P110, P111, P112, P200, P201, P209, P210, P211, P219, P90, P910, P916, P9160, P9161, P9162, P9163, P9181 |
| Hypoxic Ischaemic Encephalopathy | P916, P9160, P9163, P9162, P9161, P910, P9181 |
| Seizures | P90 |
| Intraventricular Haemorrhage | P520, P521, P522, P523, P524, P525, P528, P529 |
| Failure to progress | O620, O621, O622, O624, O628, O629, O630, O631, O632, O639, O640, O650, O651, O652, O653, O654, O662, O664, O668, O669 |
| Birth Trauma | P100, P101, P104, P108, P111, P112, P113, P120, P121, P122, P123, P124, P128, P129, P131, P133, P134, P140, P143, P152, P153, P154, P158, P159, P110, P115, P130, P132, P138, P139, P141, P150, P155, P103, P119, P102, P109, P156, P148, S020, S021, S022, P142, P151, S059, S065 |
| Brachial Plexus Trauma | P140, P143, P141 |
